# The Effect of *SF3B1* Mutation on the DNA Damage Response and Nonsense-Mediated mRNA Decay in Cancer

**DOI:** 10.3389/fonc.2020.609409

**Published:** 2021-01-29

**Authors:** Alexander C. Leeksma, Ingrid A. M. Derks, M. Haidar Kasem, Emine Kilic, Annelies de Klein, Martine J. Jager, Arjan A. van de Loosdrecht, Joop H. Jansen, Veronika Navrkalova, Laura M. Faber, Nadja Zaborsky, Alexander Egle, Thorsten Zenz, Sarka Pospisilova, Omar Abdel-Wahab, Arnon P. Kater, Eric Eldering

**Affiliations:** ^1^ Department of Hematology, Amsterdam University Medical Centers, Location AMC, University of Amsterdam, Amsterdam, Netherlands; ^2^ Department of Experimental Immunology, Amsterdam University Medical Centers, Location AMC, University of Amsterdam, Amsterdam, Netherlands; ^3^ Lymphoma and Myeloma Center Amsterdam (LYMMCARE), Cancer Center Amsterdam (CCA) and Amsterdam Infection and Immunity Institute (AIII), Amsterdam, Netherlands; ^4^ Translational Functional Cancer Genomics, National Center for Tumor Diseases (NCT) and German Cancer Research Center (DKFZ), Heidelberg, Germany; ^5^ Department of Ophthalmology and Clinical Genetics Erasmus MC, Rotterdam, Netherlands; ^6^ Department of Ophthalmology, LUMC, Leiden, Netherlands; ^7^ Department of Hematology, Amsterdam University Medical Centers, Location VUMC, Amsterdam, Netherlands; ^8^ Laboratory of Hematology, Department Laboratory Medicine, Radboud University Medical Center, Nijmegen, Netherlands; ^9^ Center of Molecular Biology and Gene Therapy, Department of Internal Medicine-Hematology and Oncology, University Hospital Brno and Center of Molecular Medicine, CEITEC, Masaryk University, Brno, Czechia; ^10^ Department of Internal Medicine, Rode Kruis Ziekenhuis, Beverwijk, Netherlands; ^11^ Department of Internal Medicine III with Haematology, Medical Oncology, Haemostaseology, Infectiology and Rheumatology, Oncologic Center, Paracelsus Medical University, Salzburg, Austria; ^12^ Department of Internal Medicine III with Haematology, Salzburg Cancer Research Institute—Laboratory for Immunological and Molecular Cancer Research (SCRI-LIMCR), Salzburg, Austria; ^13^ Department of Internal Medicine III with Haematology, Cancer Cluster Salzburg, Salzburg, Austria; ^14^ Department of Oncology and Haematology, University Hospital and University of Zurich, Zurich, Switzerland; ^15^ Human Oncology and Pathogenesis Program and Leukemia Service, Department of Medicine, Memorial Sloan Kettering Cancer Center, New York, NY, United States

**Keywords:** SF3B1, DNA damage response, splicing, nonsense-mediated mRNA decay, apoptosis

## Abstract

Recurrent mutations in splicing factor 3B subunit 1 (*SF3B1*) have been identified in several malignancies and are associated with an increased expression of 3’ cryptic transcripts as a result of alternative branchpoint recognition. A large fraction of cryptic transcripts associated with *SF3B1* mutations is expected to be sensitive for RNA degradation *via* nonsense-mediated mRNA decay (NMD). Several studies indicated alterations in various signaling pathways in SF3B1-mutated cells, including an impaired DNA damage response (DDR) in chronic lymphocytic leukemia (CLL). In this study, we investigated isogenic cell lines and treatment naïve primary CLL samples without any *TP53* and/or *ATM* defect, and found no significant effects of *SF3B1* mutations on the ATM/p53 response, phosphorylation of H2AX and sensitivity to fludarabine. Cryptic transcripts associated with *SF3B1* mutation status were observed at relatively low levels compared to the canonical transcripts and were validated as target for mRNA degradation *via* NMD. Expression of cryptic transcripts increased after NMD inhibition. In conclusion, our results confirm involvement of NMD in the biological effects of *SF3B1* mutations. Further studies may elucidate whether *SF3B1*-mutant patients could benefit from NMD modulatory agents.

## Introduction

Splicing factor 3B subunit 1 (*SF3B1*) is frequently mutated in different malignancies. In chronic lymphocytic leukemia (CLL), different studies reported a lower incidence (5–11%) of *SF3B1* mutations at diagnosis, which increased with therapy resistance to 15–20% (1, 2), and an association with poor prognosis (3, 4). In various other cancers, notably myelodysplastic syndrome (MDS; 25–30%) (5–7) and uveal melanoma (UM; 10–21%) (8–10), heterozygous *SF3B1* mutations are also highly prevalent. *SF3B1* mutations cause altered splice branchpoint recognition which results in increased 3’ cryptic splicing, and concomitant frameshifts (11, 12). Alternative transcripts with a premature termination codon (PTC) ≥50–55 nucleotides before the last exon-exon junction are normally targeted for degradation *via* nonsense-mediated mRNA decay (NMD) (11, 13). Consequently, a substantial fraction of the *SF3B1-*associated cryptic transcripts is expected to be NMD-sensitive. The pathobiology of *SF3B1* mutations is of interest because the (defective) splicing machinery might be a therapeutic target (14). Homozygous splicing factor mutations are not observed and mutations in splicing factor genes show mutual exclusivity (15). The enhanced sensitivity of *SF3B1*-mutated cells to the splicing inhibitor H3B-8800 which is currently tested in phase I clinical trials (16) is in agreement with a therapeutic window of splicing factor inhibitors. Various studies have described the effects of *SF3B1* mutations on alternative branchpoint recognition and indicated alterations in several signaling pathways including the DNA damage response (17–19), telomere maintenance (18), NF-kB (15, 20), NOTCH1 (18) and MYC signaling (21), but there is no clear view or consensus on the resulting pathological mechanism(s).

Here, we expand on our earlier observation that *SF3B1* mutations in CLL associate with an altered response to DNA damage (DDR), with certain aspects resembling an ATM defect (17). Outcomes of this previous study included effects of *SF3B1* mutations on the ATM/p53 response after irradiation, higher phosphorylation of variant histone H2AX on Ser139 [γH2AX; a marker for DNA double stranded breaks (22)] at baseline and in response to irradiation, and a decreased sensitivity to fludarabine (17). To gain more insight into the underlying pathobiological mechanism of *SF3B1* mutations, we now investigated isogenic cell lines and an additional cohort of treatment-naïve primary CLL samples without a confounding *TP53* and/or *ATM* defect. Secondly, we analyzed the effect of NMD on *SF3B1-*associated cryptic transcripts in various primary cancer cells and isogenic cell lines.

## Methods

### Cell Culture

NALM-6 isogenic knock-in cell lines including different hotspot mutations in the HEAT domain of SF3B1 (parental, K700E, K666N, and H662Q) were from a previous study (11) and mutations were confirmed by Sanger sequencing. UM cell lines 92.1 (SF3B1wt) and Mel202 (SF3B1mut) were acquired from Martine de Jager (department of ophthalmology LUMC, The Netherlands). Pancreas carcinoma (PDA) cell line panc1 (SF3B1wt) was obtained from the LEXOR group (Amsterdam UMC, The Netherlands) and panc05.04 (SF3B1mut) was directly bought from ATCC and CLL cell lines PGA (SF3B1wt) and CII (SF3B1mut) were a kind gift from Tanja Stankovic (Bournemouth, UK). Cell lines were maintained in RPMI 1640 medium (Thermo Fisher Scientific, Waltham, MA, USA) with HEPES and L-glutamine (92.1, Mel202, PGA, CII, panc05.04, and NALM-6 cell lines) or IMDM (Lonza, Basel, Switzerland) with HEPES, L-glutamine (panc1), and supplemented with 10% fetal calf serum (FCS) and penicillin-streptomycin (Invitrogen) and incubated in 5% CO_2_ at 37 °C. Panc05.04 was cultured in the presence of 1% Insulin-Transferrin-Selenium (ITS -G) (Thermo Fisher Scientific, Waltham, MA, USA). Primary CLL cells were thawed and cultured in IMDM (Lonza, Basel, Switzerland) with HEPES, L-glutamine, 10% FCS, and Penicillin-Streptomycin (Invitrogen) for functional experiments and incubated in 5% CO_2_ at 37 °C.

### RNA Extraction and Quantitative Real-Time Polymerase Chain Reaction

Total RNA was isolated using the GeneEluteTM Mammalian Total RNA Miniprep kit (Sigma-Aldrich #RTN70) and cDNA was transcribed by RevertAid (Fermentas Inc., Hannover, Md #EP0451) using Random Hexamer Primers (Promega, Madison, USA #C1181) according to manufacturer’s instructions. Primers used for detection of 3’ cryptic transcripts associated with *SF3B1* mutation were designed based on results from transcriptomic analyses (12, 23) of *SF3B1* mutated cancer cells and are listed in **Supplemental Table 1**. Expression was normalized to glyceraldehyde 3-phosphate dehydrogenase (*GAPDH*) and qPCRs were performed using SYBR Green master mix (Applied Biosystems #4385617). Linear regression (LinReg) software was used for data processing. Relative expression was calculated by the comparative ΔCt method (24).

### Sequencing of *SF3B1*

Cell lines and primary cells were sequenced with Sanger or next-generation DNA sequencing at the SF3B1locus. Primary MDS, CLL and UM cells were considered as SF3B1 mutated when a mutation was detected in the HEAT domain of SF3B1 with a variant allele frequency (VAF) ≥20%. Only treatment-naïve primary CLL cells negative for *ATM* (no del11q/and or *ATM* mutation) and *TP53* defects (no del17p and/or *TP53* mutation), at date of sampling were included for functional analysis of the DDR in *SF3B1* mutated samples. Patients characteristics and results of mutation analyses of samples used in this study are listed in **Supplemental Tables 2–5**.

### Reverse Transcriptase Multiplex Ligation Dependent Probe Amplification

For RT-MLPA analysis cells were treated with or without irradiation (1Gy or 5Gy) and cultured for 16 h. RT-MLPA (MRC-Holland) was performed as described before, using an earlier validated RT-MLPA probe set, which includes several p53 and ATM target genes (*CD95*, *BAX*, *PUMA*, *p21*, *FDXR*, *PCNA*, *NME1*, *ACSM3*) (17). Expression was normalized to a panel of housekeeping genes.

### Western Blot Analysis

Cells were lysed in Laemmli sample buffer and western blotting was performed using standard conditions. The following antibodies were used: PUMA (Sigma-Aldrich #PRS3043), p53 (Calbiochem #OP43), serine 15 phosphorylated-p53 (Cell Signaling #9284S), MDM2 (Santa Cruz #sc-965), KAP (Cell Signaling #5868), serine 824 phosphorylated-KAP (Cell Signaling #4127), p21 (Cell Signaling #2947), and β-actin (Santa Cruz #sc-1616). IRDye 800CW Goat anti-Rabbit IgG (LI-COR #926-32211), IRDye 800CW Donkey anti-Goat (LI-COR #926-32214), IRDye 680LT Donkey anti-Goat (LI-COR #926-32224), IRDye 680LT Goat anti-Mouse IgG (LI-COR #926-68020). Protein expression was quantified with Odyssey software (Li-Cor Biosciences) and corrected for the expression of β-actin.

### Apoptosis Induction by Fludarabine or Doxorubicin

Cells were cultured in the presence of fludarabine (Sigma-Aldrich #F2773) for 48 h, and doxorubicin (Selleckhem #S1208) for 24 h at indicated concentrations. Apoptosis was measured by flow cytometry. Cells were stained with 0.01 μM of the viability dye Dihexyloxacarbocyanine Iodide (DiOC6, Molecular Probes #D-273) for 20 min at 37 °C and prior to analysis, TO-PRO-3 (Thermofisher Scientific #T3605) was added as a marker for cell death. Signals were measured on a FACS Calibur (BD). Specific cell death was calculated as [(% apoptosis treated cells - % apoptosis untreated cells)/% viable untreated cells]*100. Flow cytometry data were analyzed using FlowJo software (Treestar, Ashland, OR, USA).

### γH2AX and CD95 Expression

Expression of γH2AX was measured using flow cytometry. Cells were irradiated (1Gy or 5Gy) and at indicated times, cells were permeabilized (Foxp3 staining kit; eBioscience) and stained using the following antibodies: isotype-AF488 (BD Biosciences #557782) or γH2AX-AF488 (phosphorylated-H2AX-ser-139; Cell Signaling #9719S). CD95 expression on NALM-6 cells was determined by flow cytometry using anti-CD95-FITC (BD biosciences #555673) following irradiation (1Gy or 5Gy) and 16 h culturing. Data were normalized for isotype control (isotype-AF488).

### Statistical Analysis

Analyses were performed using Graphpad Prism software version 8. (Graphpad, La Jolla, CA, USA). Kruskal–Wallis test with Dunn’s multiple comparison *post hoc* analysis was used for analysis of RT-MLPA data. A two-sided Mann–Whitney U test was used to identify differences between two groups. For apoptotic responses with >2 groups, one-way ANOVA with Dunnet’s *post hoc* test was used. P-values <0.05 were considered statistically significant.

## Results

First, we studied the ATM/p53 response in isogenic NALM-6 cells with heterozygous *SF3B1* mutations (11). Confirmation of altered SF3B1 function in three *SF3B1*-mutated NALM-6 cell lines against their parental cell line was obtained through increased expression of the *SF3B1-*associated cryptic transcripts of *ATM*, *FOXP1* and *TTI1* (**Supplemental Figure S1A**).These transcripts were previously reported to be increased in SF3B1 mutated cells (12, 23, 25), and were considered as signature genes that might also be linked with pathobiological consequences. Various aspects of DDR functionality (17) were investigated: 1) irradiation (IR) followed by quantification of ATM/p53 target genes by RT-MLPA and analysis of proteins by western blot, 2) ATM functionality *via* KAP phosphorylation on Ser824, 3) γH2AX following IR, and 4) treatment with DNA damaging agents fludarabine and doxorubicin, followed by assessment of the apoptotic response by flow cytometry. In all of these aspects, the three *SF3B1-*mutated NALM-6 cell lines behaved identical to the parental cells (**Supplemental Figures S1B-C, S2-4**). Influence of cell cycle status was investigated with the cyclin-dependent kinase inhibitor palbociclib. Palbociclib induced growth arrest without cell death induction, but did not influence the ATM/p53 response following IR (**Supplemental Figure S5**).

**Figure 1:**
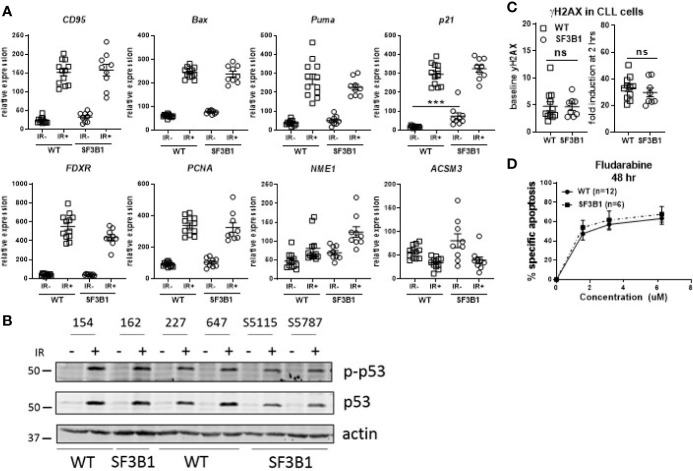
Analysis of DDR response in treatment-naïve *SF3B1-*mutated CLL cells. **(A)** Results of RT-MLPA with specific probes for the detection of ATM/p53 target genes. Relative expression is shown of treatment-naïve *SF3B1*wt (WT, n = 12) and *SF3B1*mut (SF3B1, n = 9) primary CLL cells -/+ IR (5Gy) and 16 h culturing. Data is represented as mean ± SEM. Significance was determined by Kruskal-Wallis test with Dunn’s multiple comparison *post hoc* analysis, ***p < 0.001. **(B)** Effects of IR (5Gy) followed by 16 h culturing on p-p53, p53, and β-actin measured by western blotting. **(C)** Formation of γH2AX measured by flow cytometry at baseline (left) and after irradiation (5Gy) and 2 h incubation (right). Data were normalized for isotype control and represented as mean ± SEM. ns, not significant. **(D)** CLL cells of *SF3B1*wt (n = 12) and *SF3B1*mut (n = 8) patients were treated with different concentrations of fludarabine as indicated. Cell death was assessed by DIOC_6_/TO-PRO-3 staining and calculated as described in material and methods. Error bars are ± SEM. No significant differences were observed (Mann-Whitney).

Since our earlier studied CLL cohort contained a mix of untreated and chemotherapy-treated CLL patients, we next analyzed treatment-naïve primary CLL cells harboring *SF3B1* mutations (median VAF of 41.8%; **Supplemental Table S2**) for potential effects on the DDR using the same set of assays as applied previously (17). RT-MLPA revealed a significantly increased p21 mRNA in non-irradiated *SF3B1-*mutated CLL cells (*p* < 0.001; **Figure 1A**). This is in accordance with a recently identified link of *SF3B1* mutations with senescence and increased p21 protein levels (19). Non-irradiated *SF3B1-*mutated CLL cells showed a trend towards a higher expression of *ACSM3* with large variation between patients, which could not be linked with VAF of the mutation. Also, the response of ATM target genes *ACSM3* and *NME1* to IR was not affected by *SF3B1* mutation status (**Figure 1A**) (17). Identical effects of wild type (WT) and *SF3B1-*mutated samples in response to IR were observed in this cohort (**Figure 1A**). This was unlike the previous data on the mixed treatment cohort, where differences between WT, and *SF3B1-*mutated samples were apparent (17). Levels of Ser15 phosphorylated p53 and p53 (**Figure 1B**), γH2AX baseline/formation (**Figure 1C**) and sensitivity to fludarabine (**Figure 1D**) appeared unaffected in treatment-naïve *SF3B1-*mutated CLL cases. In summary, we could not detect mechanistic clues relating to a potential link between *SF3B1* mutation and altered DDR responses, using isogenic cell lines and a treatment-naïve CLL cohort.

**Figure 2:**
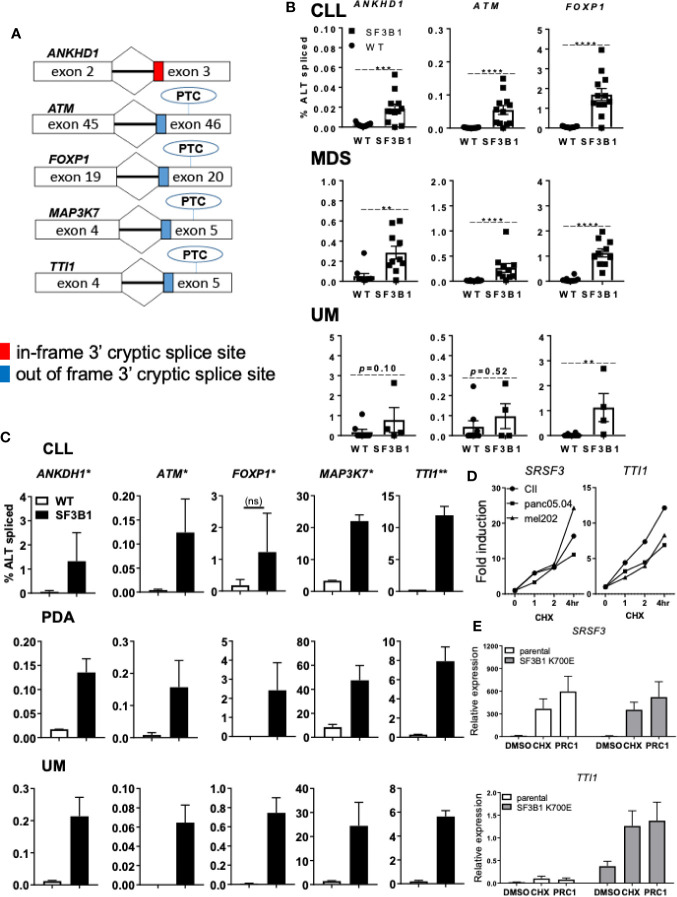
Levels of *SF3B1-*associated 3’ cryptic transcripts are often low and sensitive nonsense-mediated mRNA decay. **(A)** Schematic overview of five 3’ cryptic transcripts associated with *SF3B1* mutation and the effect of alternative splicing on the introduction of a premature termination codon (PTC) in these transcripts. **(B)** Percentage ALT spliced (percentage cryptically spliced compared to the canonical transcript; % ALT spliced; y-axis) for qPCR analysis of *ANKHD1*, *ATM*, and *FOXP1* in primary CLL cells divided in *SF3B1*wt (n = 15) and *SF3B1*mut (n = 12), myelodysplastic syndrome (MDS) divided in *SF3B1*wt (n = 10) and *SF3B1*mut (n = 10) and uveal melanoma (UM) patients divided in *SF3B1*wt (n = 8) and *SF3B1*mut (n = 4). Primers specific for the 3’ cryptic transcript and the canonical transcript were used. Bars represent mean ± SEM. Significant differences are presented as *p < 0.05, **p < 0.01, ***p < 0.001, ****p < 0.0001 (Mann-Whitney U test). **(C)** Percentage ALT spliced was calculated for *ANKHD1*, *ATM* and *FOXP1*, *MAP3K7* and *TTI1* in the CLL cell lines PGA (*SF3B1*wt) and CII (*SF3B1*mut), pancreas carcinoma (PDA) cell lines panc1 (*SF3B1*wt) and panc05.04 (*SF3B1*mut) and UM cell lines 92.1 (*SF3B1*wt) and mel202 (*SF3B1*mut). Bars represent mean ± SEM of at least two independent experiments, three for *ANKHD1* in mel202 and six for *FOXP1* in mel202). **(D)** Fold induction of NMD-sensitive transcript of *SRSF3* (left) and cryptic transcript of *TTi1* (right) 1, 2, and 4 h after cycloheximide (CHX; 100 μg/ml) treatment in CLL, PDA and UM cell lines -/+ *SF3B1* mutation. **(E)** Relative expression of NMD-sensitive transcript of *SRSF3* (above) and cryptic transcript of *TTI1* (below) 6 h after DMSO, 100 μg/ml CHX or SMG1 inhibition by 1 μM pyrimidine related compound 1 (PRC1) in isogenic NALM-6 cells -/+ *SF3B1* K700E mutation. Bars represent mean ± SEM of three independent experiments.

Another possible pathological mechanism is increased expression of *SF3B1* associated 3’ cryptic transcripts at the expense of the canonical mRNA and protein, as reported earlier (11). In addition, *SRSF2* mutations have been shown to affect both splicing and NMD, suggesting a role for NMD in the pathogenic effect of splicing factor mutations (26). Five cryptically spliced genes (*ANKHD1*, *ATM*, *FOXP1*, *MAP3K7*, and *TTI1*; **Figure 2A**), identified in previous transcriptomic analyses in *SF3B1-*mutated patients (12, 15, 18), were analyzed in different cancer types. Percentages of cryptic transcripts versus canonical transcripts were quantified in primary material from genotyped CLL (**Supplemental Table S3**), MDS (**Supplemental Table S4**) and UM (**Supplemental Table S5**), and in cancer cell lines from different origin -/+ *SF3B1* mutation (**Figures 2B, C**, respectively). Increased expression of *SF3B1-*associated transcripts was indeed observed in all *SF3B1-*mutated cells compared to *SF3B1* WT cells in primary cancer cells and cancer cell lines. Ratios of cryptic versus canonical transcripts were gene-specific and differed between the investigated cancer cells. Cryptic transcripts were mostly present at 10–1,000-fold lower levels than the canonical transcripts, only for MAP3K7 it reached appreciable, though still modest levels.

NMD and its potential altered function/contribution in cancer has recently become of interest as a therapeutic target (13). To explore the effect of NMD on the expression of *SF3B1-*associated cryptic transcripts, we inhibited NMD in different cell lines -/+ *SF3B1* mutation. Cells were treated with the translation inhibitor cycloheximide (CHX), known for its ability to inhibit NMD (11), or pyrimidine related compound 1 (PRC1), a specific inhibitor of the PI3K related kinase SMG1 which regulates NMD activity *via* phosphorylation of UPF1 (27). NMD inhibition with CHX was confirmed by analysis of an established NMD transcript of the splicing factor *SRSF3* (**Figure 2D**) (26). As expected, a rapidly increasing expression of the NMD-sensitive *SRSF3* transcript was observed after CHX treatment in *SF3B1-*mutated cells originating from various cancer types (CLL, UM and pancreatic cancer). Expression of the *SF3B1*-associated cryptic transcript of *TTI1* also increased after NMD inhibition (**Figure 2D** *)*. SMG1 inhibition with PRC1 also resulted in an increased expression of *SRSF3* and *TTI1* transcripts in *SF3B1-*mutated NALM-6 cells (**Figure 2E**). These results suggest that *SF3B1-*associated cryptic transcripts are degraded *via* NMD and suggest a link between NMD and the pathogenic effects of *SF3B1* mutations.

## Discussion

Various clinical trials have reported a negative effect of *SF3B1* mutations on survival in chemotherapy-treated CLL patients. Mutations in SF3B1 were associated with decreased survival after chlorambucil and fludarabine with and without cyclophosphamide in the UK CLL4 trial (4) and fludarabine, cyclophosphamide plus rituximab in the German CLL8 trial (3). To expand our previous study, we therefore selected treatment-naïve samples with a high VAF of *SF3B1* mutation. Our results demonstrate that *SF3B1* mutations do not directly affect the ATM/p53 response, at least not in uncompromised, untreated patients. The seeming differences between earlier reported data on a mixed cohort of patients might be explained by effects of chemotherapy; most likely this resulted in the outgrowth of cells with defects in genes other than *ATM* and/or *TP53*, while still causing a slightly defective DDR response. Therefore, we should consider that *SF3B1* mutations can affect genomic stability *via* other pathways (28), or that other factors are associated with progression of *SF3B1-*mutant CLL patients. For example, mutations in splicing factors in MDS were linked with augmented R-loops and alternative transcripts were observed in genes involved in the suppression of R-loop formation (29). In addition, various altered transcripts resulting from *SF3B1* mutation were linked with diverse signaling pathways; decreased MAP3K7 expression leading to increased NF-kB activity (15), decreased expression of the uveal melanoma tumor suppressor gene BRD9 resulting in disruption of the non-canonical BAF chromatin-remodeling complex (30), decreased phosphatase 2A subunit PPP2R5A leading to MYC stability (21), and an alternative transcript of *DVL2* was linked to overexpression of NOTCH1 in CLL (18). As hundreds of genes are associated with increased levels of cryptically spliced transcripts in *SF3B1* mutants (11, 12, 15, 18), it is to be expected that *SF3B1* mutations may have widespread effects on cancer cells. Indeed, differences in numerous pathways have been identified in a recent transcriptomic analysis (18). We observed a distinct increase in expression of cryptic transcripts associated with *SF3B1* mutations. Cryptic transcripts were mostly at 10–1,000-fold lower levels than the canonical transcripts, which may in most cases decrease the likelihood this would reach pathological levels. Yet, such transcripts could be augmented upon NMD inhibition, a finding that warrants further study. Hypothetically, the increased expression of cryptic transcripts observed in *SF3B1* mutants could be used therapeutically as NMD inhibition might result in the presentation of tumor specific neoantigens (31).

In conclusion, our results suggest a role for NMD in the biological effects of *SF3B1* mutations and indicate that *SF3B1* mutant patients could potentially benefit from NMD modulatory agents.

## Data Availability Statement

The original contributions presented in the study are included in the article/supplementary material, further inquiries can be directed to the corresponding author/s.

## Ethics Statement

The study “B cell maligniteiten Biobank” was approved by the ethical committee Biobank Toetsing Comissie at AMC under the number METC 2013/159. Participants have local approval by their hospital RvB and local ethical committee. The patients/participants provided their written informed consent to participate in this study.

## Author Contributions

AL, AK, and EE were responsible for the conception and design of the study. AL, ID, HK, EK, MJ, AdK, AL, JJ, VN, RK, NZ, AE, TZ, SP, OA-W, AK, and EE were involved in the analysis and interpretation of the data. AL, ID, and HK perfomed statistical analyses, made the figures, and performed the experiments. LF contributed clinical CLL samples. All authors contributed to the article and approved the submitted version.

## Funding

AL is supported by the van der Laan Foundation. VN and SP were supported by projects MEYS CR CEITEC2020 LQ1601, MZCR-RVO 65269705, and GA CR 19-15737S. Contribution of AE was supported by Austrian FWF grant (ERA-NET TRANSCAN-2 program JTC 2014–project FIRE-CLL; I2795-B28).

## Conflict of Interest

The authors declare that the research was conducted in the absence of any commercial or financial relationships that could be construed as a potential conflict of interest.
