# Supplementary material for: The Effect of SF3B1 Mutation on the DNA Damage Response and Nonsense-Mediated mRNA Decay in Cancer
Source: Front Oncol. 2021 Jan 29;10:609409. doi: 10.3389/fonc.2020.609409 (PMC7880055; doi:10.3389/fonc.2020.609409)
Supplement: Supplementary file 1 [file Presentation_1.pptx]

## Slide 1
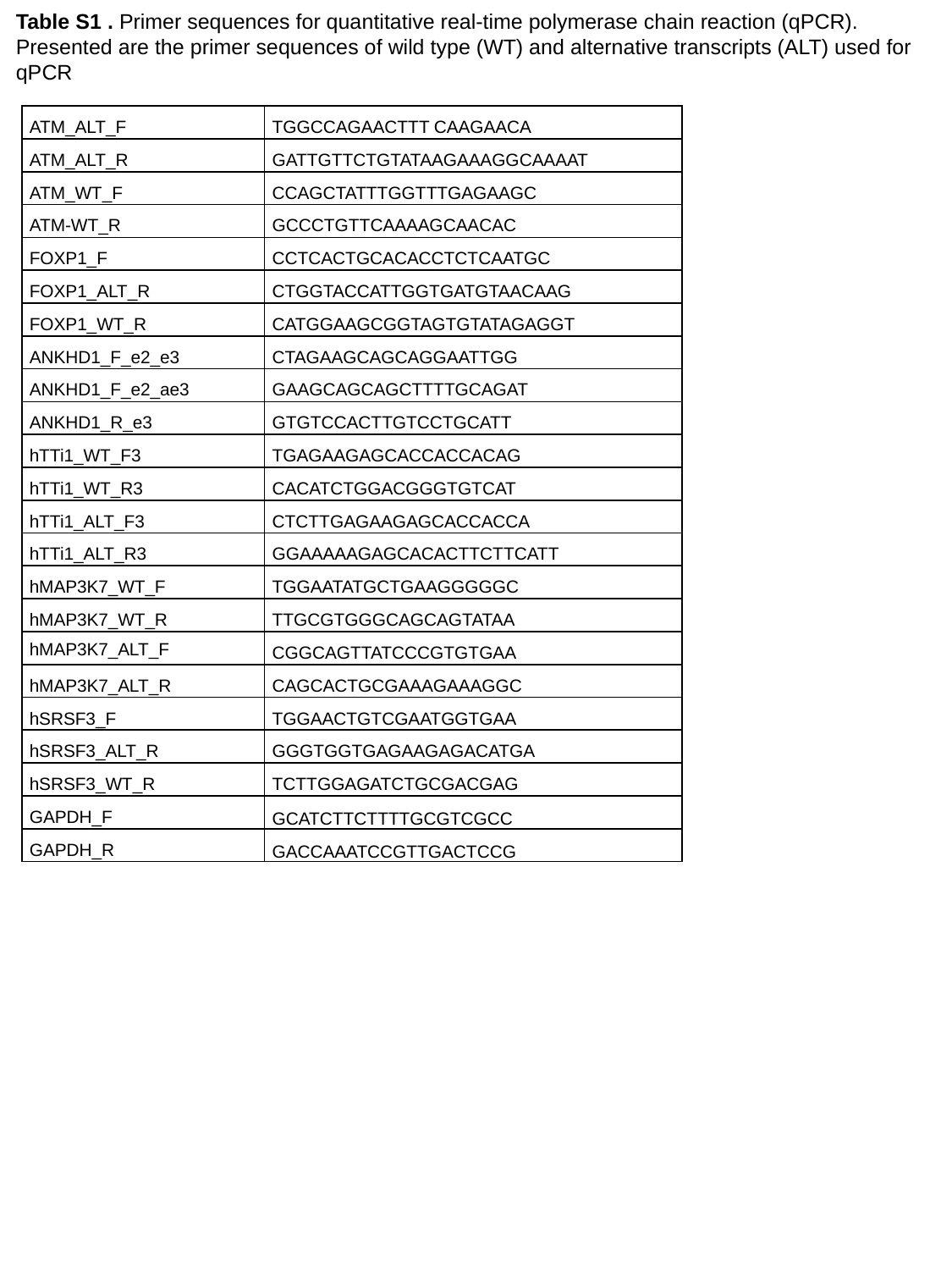

Table S1 . Primer sequences for quantitative real-time polymerase chain reaction (qPCR). Presented are the primer sequences of wild type (WT) and alternative transcripts (ALT) used for qPCR
| ATM\_ALT\_F | TGGCCAGAACTTT CAAGAACA |
| --- | --- |
| ATM\_ALT\_R | GATTGTTCTGTATAAGAAAGGCAAAAT |
| ATM\_WT\_F | CCAGCTATTTGGTTTGAGAAGC |
| ATM-WT\_R | GCCCTGTTCAAAAGCAACAC |
| FOXP1\_F | CCTCACTGCACACCTCTCAATGC |
| FOXP1\_ALT\_R | CTGGTACCATTGGTGATGTAACAAG |
| FOXP1\_WT\_R | CATGGAAGCGGTAGTGTATAGAGGT |
| ANKHD1\_F\_e2\_e3 | CTAGAAGCAGCAGGAATTGG |
| ANKHD1\_F\_e2\_ae3 | GAAGCAGCAGCTTTTGCAGAT |
| ANKHD1\_R\_e3 | GTGTCCACTTGTCCTGCATT |
| hTTi1\_WT\_F3 | TGAGAAGAGCACCACCACAG |
| hTTi1\_WT\_R3 | CACATCTGGACGGGTGTCAT |
| hTTi1\_ALT\_F3 | CTCTTGAGAAGAGCACCACCA |
| hTTi1\_ALT\_R3 | GGAAAAAGAGCACACTTCTTCATT |
| hMAP3K7\_WT\_F | TGGAATATGCTGAAGGGGGC |
| hMAP3K7\_WT\_R | TTGCGTGGGCAGCAGTATAA |
| hMAP3K7\_ALT\_F | CGGCAGTTATCCCGTGTGAA |
| hMAP3K7\_ALT\_R | CAGCACTGCGAAAGAAAGGC |
| hSRSF3\_F | TGGAACTGTCGAATGGTGAA |
| hSRSF3\_ALT\_R | GGGTGGTGAGAAGAGACATGA |
| hSRSF3\_WT\_R | TCTTGGAGATCTGCGACGAG |
| GAPDH\_F | GCATCTTCTTTTGCGTCGCC |
| GAPDH\_R | GACCAAATCCGTTGACTCCG |

## Slide 2
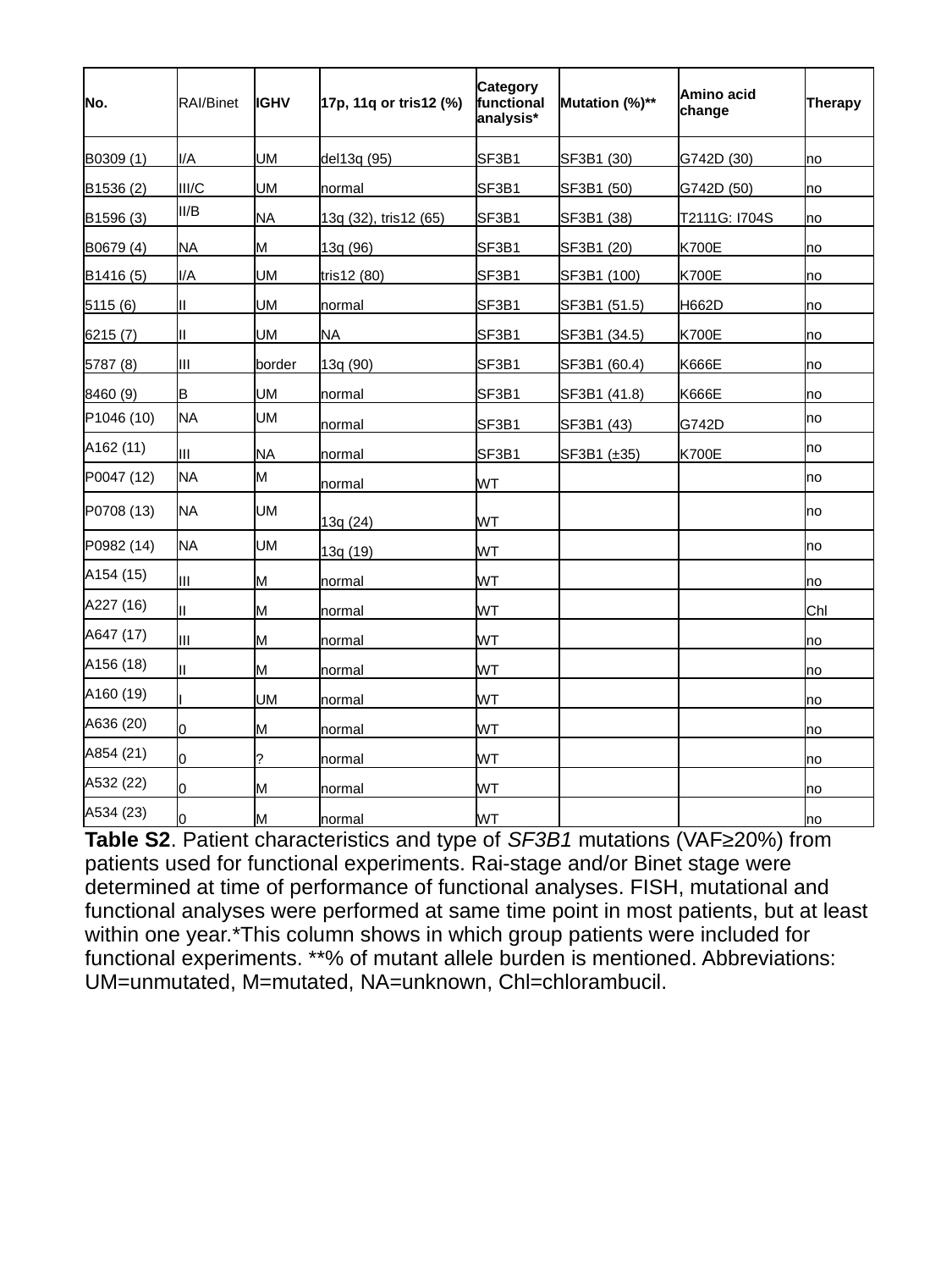

| No. | RAI/Binet | IGHV | 17p, 11q or tris12 (%) | Category functional analysis\* | Mutation (%)\*\* | Amino acid change | Therapy |
| --- | --- | --- | --- | --- | --- | --- | --- |
| B0309 (1) | I/A | UM | del13q (95) | SF3B1 | SF3B1 (30) | G742D (30) | no |
| B1536 (2) | III/C | UM | normal | SF3B1 | SF3B1 (50) | G742D (50) | no |
| B1596 (3) | II/B | NA | 13q (32), tris12 (65) | SF3B1 | SF3B1 (38) | T2111G: I704S | no |
| B0679 (4) | NA | M | 13q (96) | SF3B1 | SF3B1 (20) | K700E | no |
| B1416 (5) | I/A | UM | tris12 (80) | SF3B1 | SF3B1 (100) | K700E | no |
| 5115 (6) | II | UM | normal | SF3B1 | SF3B1 (51.5) | H662D | no |
| 6215 (7) | II | UM | NA | SF3B1 | SF3B1 (34.5) | K700E | no |
| 5787 (8) | III | border | 13q (90) | SF3B1 | SF3B1 (60.4) | K666E | no |
| 8460 (9) | B | UM | normal | SF3B1 | SF3B1 (41.8) | K666E | no |
| P1046 (10) | NA | UM | normal | SF3B1 | SF3B1 (43) | G742D | no |
| A162 (11) | III | NA | normal | SF3B1 | SF3B1 (±35) | K700E | no |
| P0047 (12) | NA | M | normal | WT | | | no |
| P0708 (13) | NA | UM | 13q (24) | WT | | | no |
| P0982 (14) | NA | UM | 13q (19) | WT | | | no |
| A154 (15) | III | M | normal | WT | | | no |
| A227 (16) | II | M | normal | WT | | | Chl |
| A647 (17) | III | M | normal | WT | | | no |
| A156 (18) | II | M | normal | WT | | | no |
| A160 (19) | I | UM | normal | WT | | | no |
| A636 (20) | 0 | M | normal | WT | | | no |
| A854 (21) | 0 | ? | normal | WT | | | no |
| A532 (22) | 0 | M | normal | WT | | | no |
| A534 (23) | 0 | M | normal | WT | | | no |
| Table S2. Patient characteristics and type of SF3B1 mutations (VAF≥20%) from patients used for functional experiments. Rai-stage and/or Binet stage were determined at time of performance of functional analyses. FISH, mutational and functional analyses were performed at same time point in most patients, but at least within one year.\*This column shows in which group patients were included for functional experiments. \*\*% of mutant allele burden is mentioned. Abbreviations: UM=unmutated, M=mutated, NA=unknown, Chl=chlorambucil. | | | | | | | |

## Slide 3
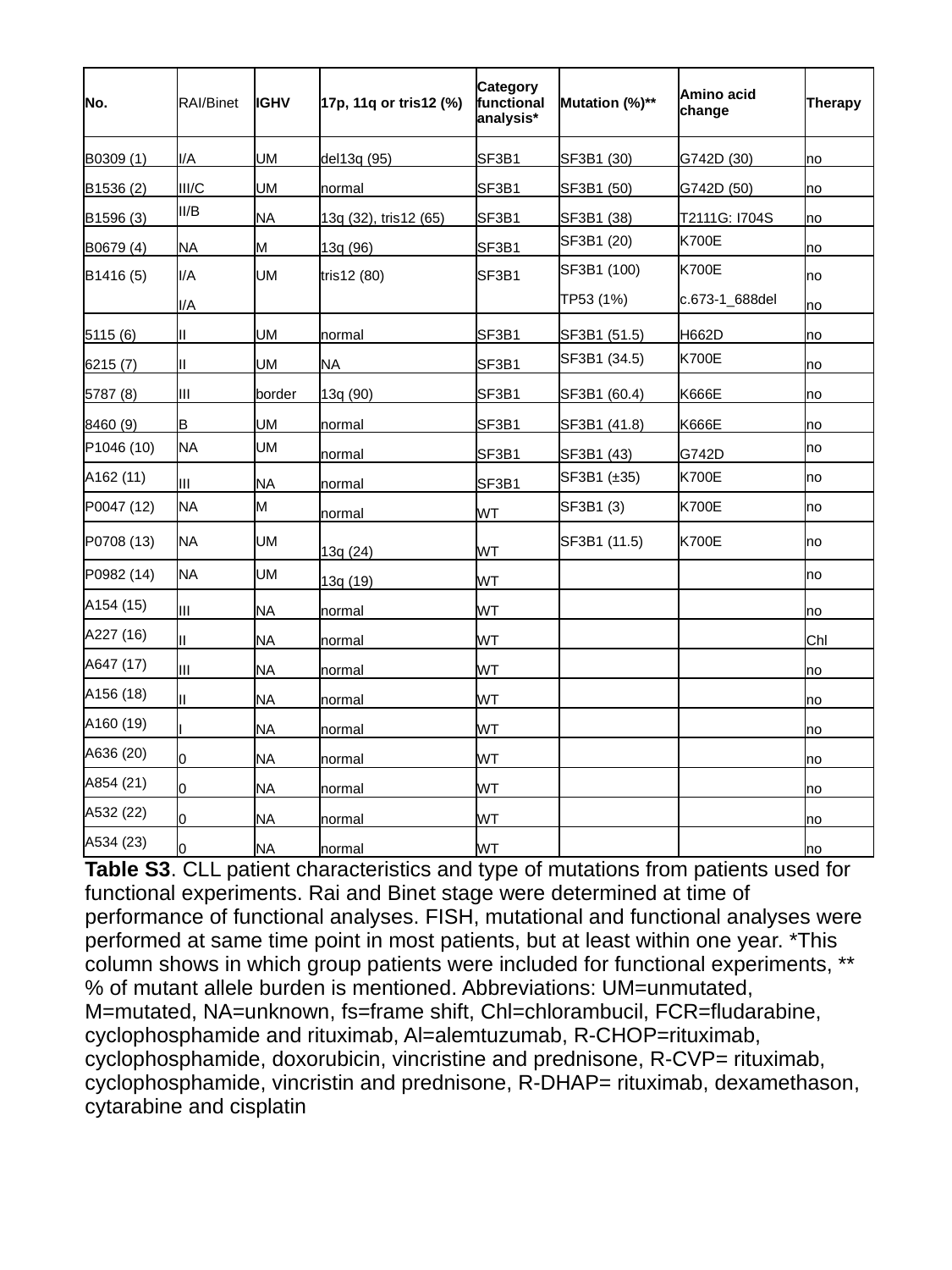

| No. | RAI/Binet | IGHV | 17p, 11q or tris12 (%) | Category functional analysis\* | Mutation (%)\*\* | Amino acid change | Therapy |
| --- | --- | --- | --- | --- | --- | --- | --- |
| B0309 (1) | I/A | UM | del13q (95) | SF3B1 | SF3B1 (30) | G742D (30) | no |
| B1536 (2) | III/C | UM | normal | SF3B1 | SF3B1 (50) | G742D (50) | no |
| B1596 (3) | II/B | NA | 13q (32), tris12 (65) | SF3B1 | SF3B1 (38) | T2111G: I704S | no |
| B0679 (4) | NA | M | 13q (96) | SF3B1 | SF3B1 (20) | K700E | no |
| B1416 (5) | I/A | UM | tris12 (80) | SF3B1 | SF3B1 (100) | K700E | no |
| | I/A | | | | TP53 (1%) | c.673-1\_688del | no |
| 5115 (6) | II | UM | normal | SF3B1 | SF3B1 (51.5) | H662D | no |
| 6215 (7) | II | UM | NA | SF3B1 | SF3B1 (34.5) | K700E | no |
| 5787 (8) | III | border | 13q (90) | SF3B1 | SF3B1 (60.4) | K666E | no |
| 8460 (9) | B | UM | normal | SF3B1 | SF3B1 (41.8) | K666E | no |
| P1046 (10) | NA | UM | normal | SF3B1 | SF3B1 (43) | G742D | no |
| A162 (11) | III | NA | normal | SF3B1 | SF3B1 (±35) | K700E | no |
| P0047 (12) | NA | M | normal | WT | SF3B1 (3) | K700E | no |
| P0708 (13) | NA | UM | 13q (24) | WT | SF3B1 (11.5) | K700E | no |
| P0982 (14) | NA | UM | 13q (19) | WT | | | no |
| A154 (15) | III | NA | normal | WT | | | no |
| A227 (16) | II | NA | normal | WT | | | Chl |
| A647 (17) | III | NA | normal | WT | | | no |
| A156 (18) | II | NA | normal | WT | | | no |
| A160 (19) | I | NA | normal | WT | | | no |
| A636 (20) | 0 | NA | normal | WT | | | no |
| A854 (21) | 0 | NA | normal | WT | | | no |
| A532 (22) | 0 | NA | normal | WT | | | no |
| A534 (23) | 0 | NA | normal | WT | | | no |
| Table S3. CLL patient characteristics and type of mutations from patients used for functional experiments. Rai and Binet stage were determined at time of performance of functional analyses. FISH, mutational and functional analyses were performed at same time point in most patients, but at least within one year. \*This column shows in which group patients were included for functional experiments, \*\*% of mutant allele burden is mentioned. Abbreviations: UM=unmutated, M=mutated, NA=unknown, fs=frame shift, Chl=chlorambucil, FCR=fludarabine, cyclophosphamide and rituximab, Al=alemtuzumab, R-CHOP=rituximab, cyclophosphamide, doxorubicin, vincristine and prednisone, R-CVP= rituximab, cyclophosphamide, vincristin and prednisone, R-DHAP= rituximab, dexamethason, cytarabine and cisplatin | | | | | | | |

## Slide 4
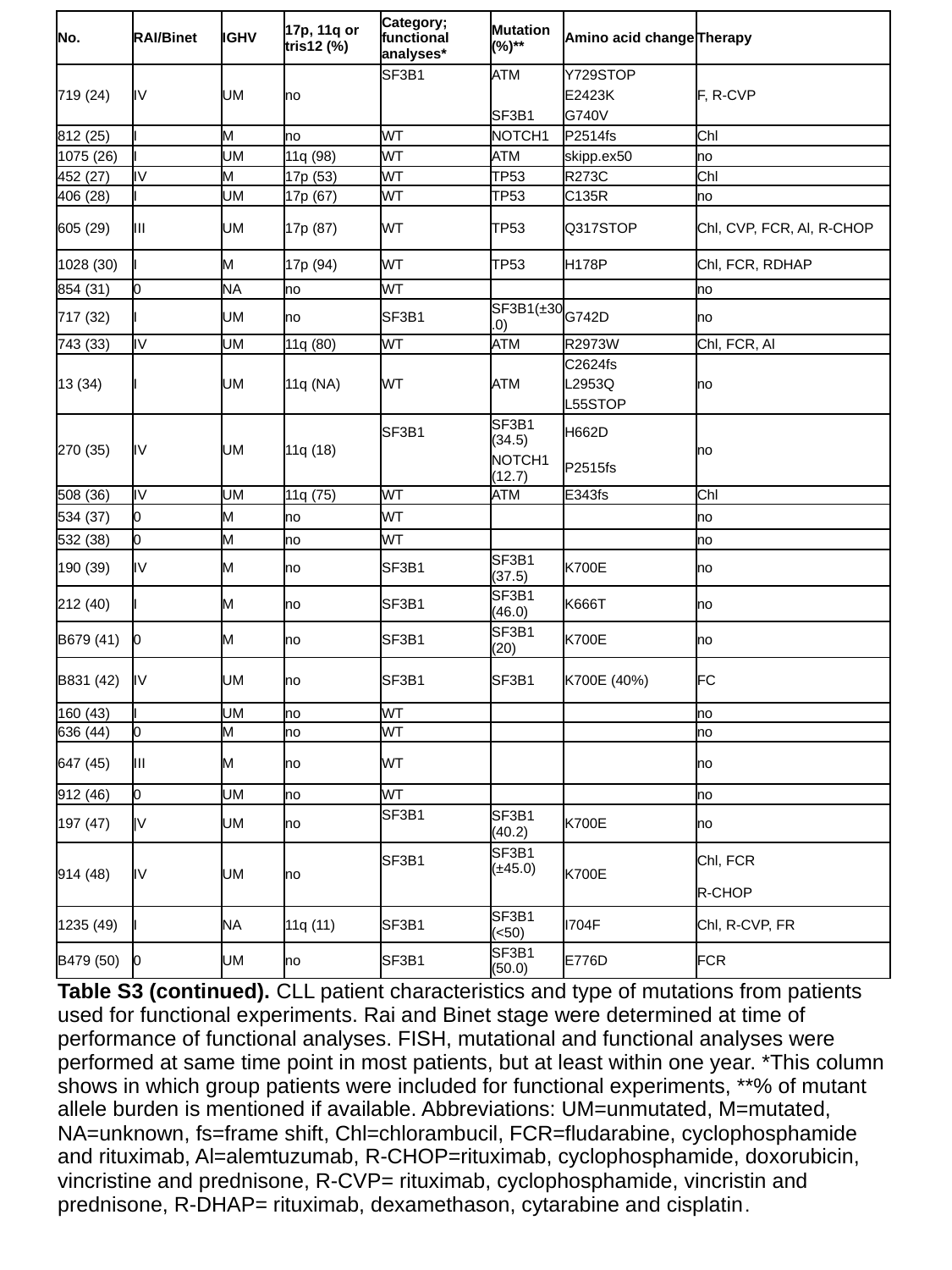

| No. | RAI/Binet | IGHV | 17p, 11q or tris12 (%) | Category; functional analyses\* | Mutation (%)\*\* | Amino acid change | Therapy |
| --- | --- | --- | --- | --- | --- | --- | --- |
| 719 (24) | IV | UM | no | SF3B1 | ATM | Y729STOP | F, R-CVP |
| | | | | | | E2423K | |
| | | | | | SF3B1 | G740V | |
| 812 (25) | I | M | no | WT | NOTCH1 | P2514fs | Chl |
| 1075 (26) | I | UM | 11q (98) | WT | ATM | skipp.ex50 | no |
| 452 (27) | IV | M | 17p (53) | WT | TP53 | R273C | Chl |
| 406 (28) | I | UM | 17p (67) | WT | TP53 | C135R | no |
| 605 (29) | III | UM | 17p (87) | WT | TP53 | Q317STOP | Chl, CVP, FCR, Al, R-CHOP |
| 1028 (30) | I | M | 17p (94) | WT | TP53 | H178P | Chl, FCR, RDHAP |
| 854 (31) | 0 | NA | no | WT | | | no |
| 717 (32) | I | UM | no | SF3B1 | SF3B1(±30.0) | G742D | no |
| 743 (33) | IV | UM | 11q (80) | WT | ATM | R2973W | Chl, FCR, Al |
| 13 (34) | I | UM | 11q (NA) | WT | ATM | C2624fs | no |
| | | | | | | L2953Q | |
| | | | | | | L55STOP | |
| 270 (35) | IV | UM | 11q (18) | SF3B1 | SF3B1 (34.5) | H662D | no |
| | | | | | NOTCH1 (12.7) | P2515fs | |
| 508 (36) | IV | UM | 11q (75) | WT | ATM | E343fs | Chl |
| 534 (37) | 0 | M | no | WT | | | no |
| 532 (38) | 0 | M | no | WT | | | no |
| 190 (39) | IV | M | no | SF3B1 | SF3B1 (37.5) | K700E | no |
| 212 (40) | I | M | no | SF3B1 | SF3B1 (46.0) | K666T | no |
| B679 (41) | 0 | M | no | SF3B1 | SF3B1 (20) | K700E | no |
| B831 (42) | IV | UM | no | SF3B1 | SF3B1 | K700E (40%) | FC |
| 160 (43) | I | UM | no | WT | | | no |
| 636 (44) | 0 | M | no | WT | | | no |
| 647 (45) | III | M | no | WT | | | no |
| 912 (46) | 0 | UM | no | WT | | | no |
| 197 (47) | |V | UM | no | SF3B1 | SF3B1 (40.2) | K700E | no |
| | | | | | | | |
| 914 (48) | IV | UM | no | SF3B1 | SF3B1 (±45.0) | K700E | Chl, FCR |
| | | | | | | | R-CHOP |
| 1235 (49) | I | NA | 11q (11) | SF3B1 | SF3B1 (<50) | I704F | Chl, R-CVP, FR |
| B479 (50) | 0 | UM | no | SF3B1 | SF3B1 (50.0) | E776D | FCR |
| Table S3 (continued). CLL patient characteristics and type of mutations from patients used for functional experiments. Rai and Binet stage were determined at time of performance of functional analyses. FISH, mutational and functional analyses were performed at same time point in most patients, but at least within one year. \*This column shows in which group patients were included for functional experiments, \*\*% of mutant allele burden is mentioned if available. Abbreviations: UM=unmutated, M=mutated, NA=unknown, fs=frame shift, Chl=chlorambucil, FCR=fludarabine, cyclophosphamide and rituximab, Al=alemtuzumab, R-CHOP=rituximab, cyclophosphamide, doxorubicin, vincristine and prednisone, R-CVP= rituximab, cyclophosphamide, vincristin and prednisone, R-DHAP= rituximab, dexamethason, cytarabine and cisplatin. | | | | | | | |

## Slide 5
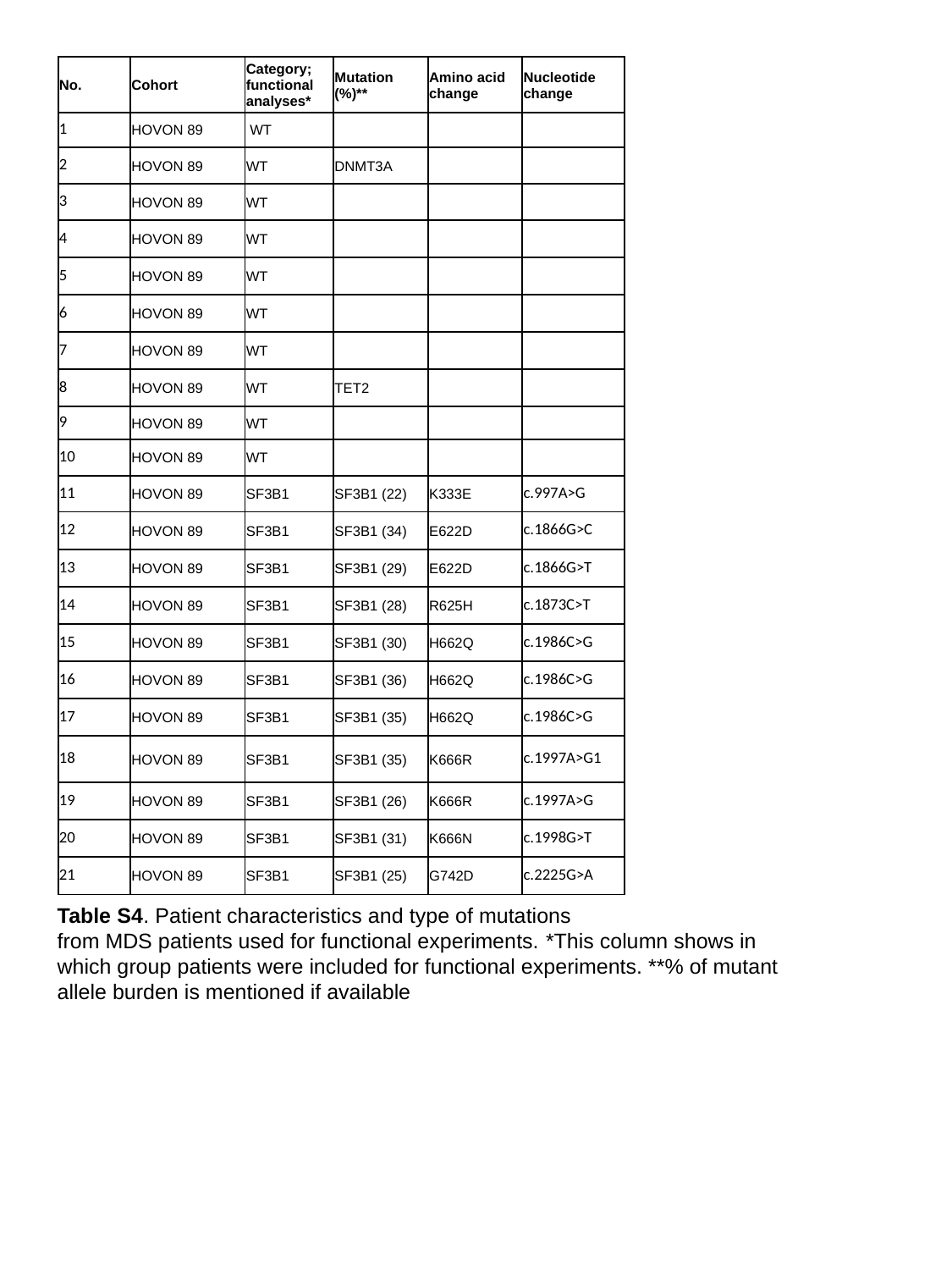

| No. | Cohort | Category; functional analyses\* | Mutation (%)\*\* | Amino acid change | Nucleotide change |
| --- | --- | --- | --- | --- | --- |
| 1 | HOVON 89 | WT | | | |
| 2 | HOVON 89 | WT | DNMT3A | | |
| 3 | HOVON 89 | WT | | | |
| 4 | HOVON 89 | WT | | | |
| 5 | HOVON 89 | WT | | | |
| 6 | HOVON 89 | WT | | | |
| 7 | HOVON 89 | WT | | | |
| 8 | HOVON 89 | WT | TET2 | | |
| 9 | HOVON 89 | WT | | | |
| 10 | HOVON 89 | WT | | | |
| 11 | HOVON 89 | SF3B1 | SF3B1 (22) | K333E | c.997A>G |
| 12 | HOVON 89 | SF3B1 | SF3B1 (34) | E622D | c.1866G>C |
| 13 | HOVON 89 | SF3B1 | SF3B1 (29) | E622D | c.1866G>T |
| 14 | HOVON 89 | SF3B1 | SF3B1 (28) | R625H | c.1873C>T |
| 15 | HOVON 89 | SF3B1 | SF3B1 (30) | H662Q | c.1986C>G |
| 16 | HOVON 89 | SF3B1 | SF3B1 (36) | H662Q | c.1986C>G |
| 17 | HOVON 89 | SF3B1 | SF3B1 (35) | H662Q | c.1986C>G |
| 18 | HOVON 89 | SF3B1 | SF3B1 (35) | K666R | c.1997A>G1 |
| 19 | HOVON 89 | SF3B1 | SF3B1 (26) | K666R | c.1997A>G |
| 20 | HOVON 89 | SF3B1 | SF3B1 (31) | K666N | c.1998G>T |
| 21 | HOVON 89 | SF3B1 | SF3B1 (25) | G742D | c.2225G>A |
Table S4. Patient characteristics and type of mutations
from MDS patients used for functional experiments. *This column shows in which group patients were included for functional experiments. **% of mutant allele burden is mentioned if available

## Slide 6
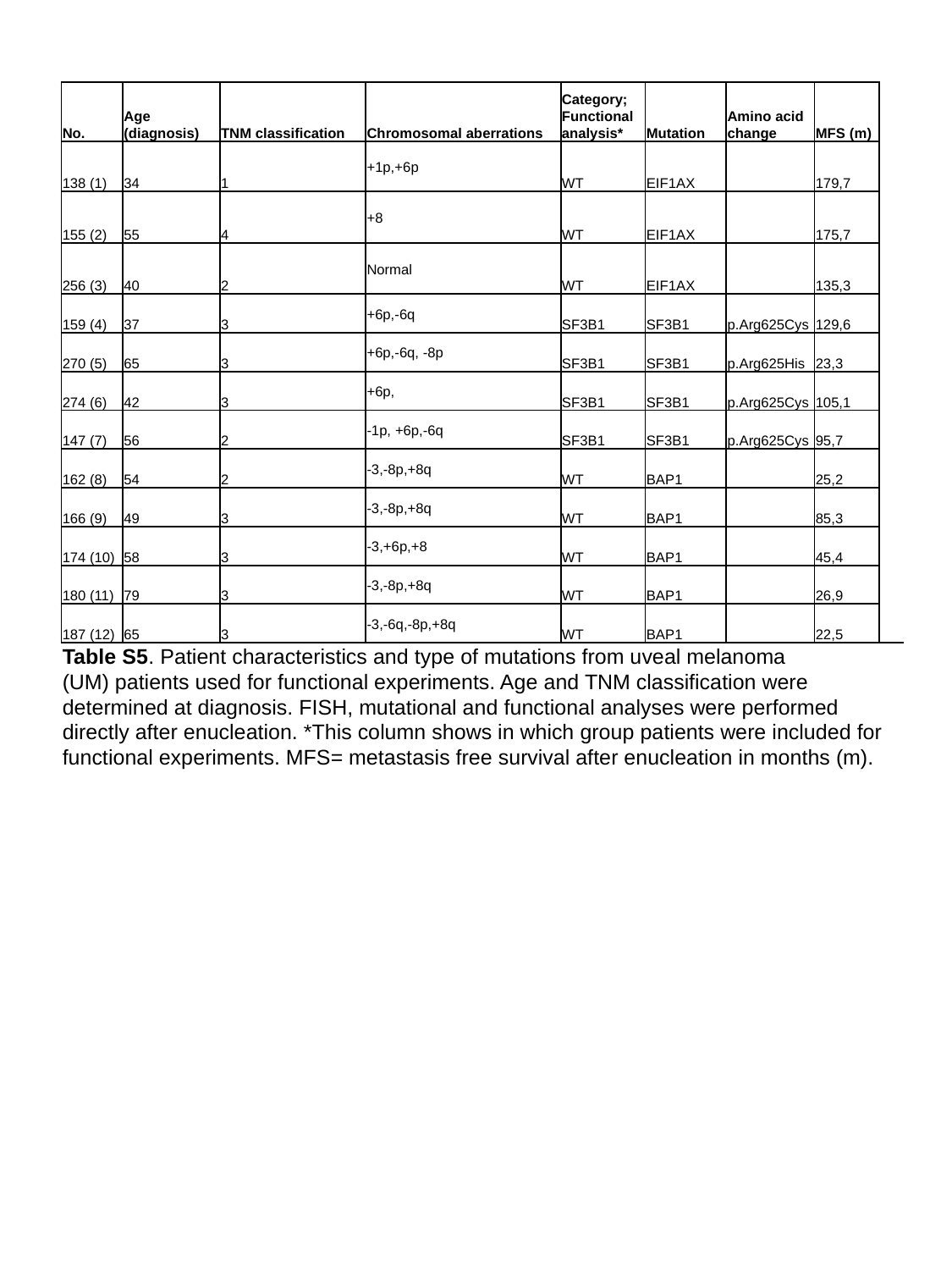

| No. | Age (diagnosis) | TNM classification | Chromosomal aberrations | Category; Functional analysis\* | Mutation | Amino acid change | MFS (m) | |
| --- | --- | --- | --- | --- | --- | --- | --- | --- |
| 138 (1) | 34 | 1 | +1p,+6p | WT | EIF1AX | | 179,7 | |
| 155 (2) | 55 | 4 | +8 | WT | EIF1AX | | 175,7 | |
| 256 (3) | 40 | 2 | Normal | WT | EIF1AX | | 135,3 | |
| 159 (4) | 37 | 3 | +6p,-6q | SF3B1 | SF3B1 | p.Arg625Cys | 129,6 | |
| 270 (5) | 65 | 3 | +6p,-6q, -8p | SF3B1 | SF3B1 | p.Arg625His | 23,3 | |
| 274 (6) | 42 | 3 | +6p, | SF3B1 | SF3B1 | p.Arg625Cys | 105,1 | |
| 147 (7) | 56 | 2 | -1p, +6p,-6q | SF3B1 | SF3B1 | p.Arg625Cys | 95,7 | |
| 162 (8) | 54 | 2 | -3,-8p,+8q | WT | BAP1 | | 25,2 | |
| 166 (9) | 49 | 3 | -3,-8p,+8q | WT | BAP1 | | 85,3 | |
| 174 (10) | 58 | 3 | -3,+6p,+8 | WT | BAP1 | | 45,4 | |
| 180 (11) | 79 | 3 | -3,-8p,+8q | WT | BAP1 | | 26,9 | |
| 187 (12) | 65 | 3 | -3,-6q,-8p,+8q | WT | BAP1 | | 22,5 | |
| Table S5. Patient characteristics and type of mutations from uveal melanoma (UM) patients used for functional experiments. Age and TNM classification were determined at diagnosis. FISH, mutational and functional analyses were performed directly after enucleation. \*This column shows in which group patients were included for functional experiments. MFS= metastasis free survival after enucleation in months (m). | | | | | | | | |

## Slide 7
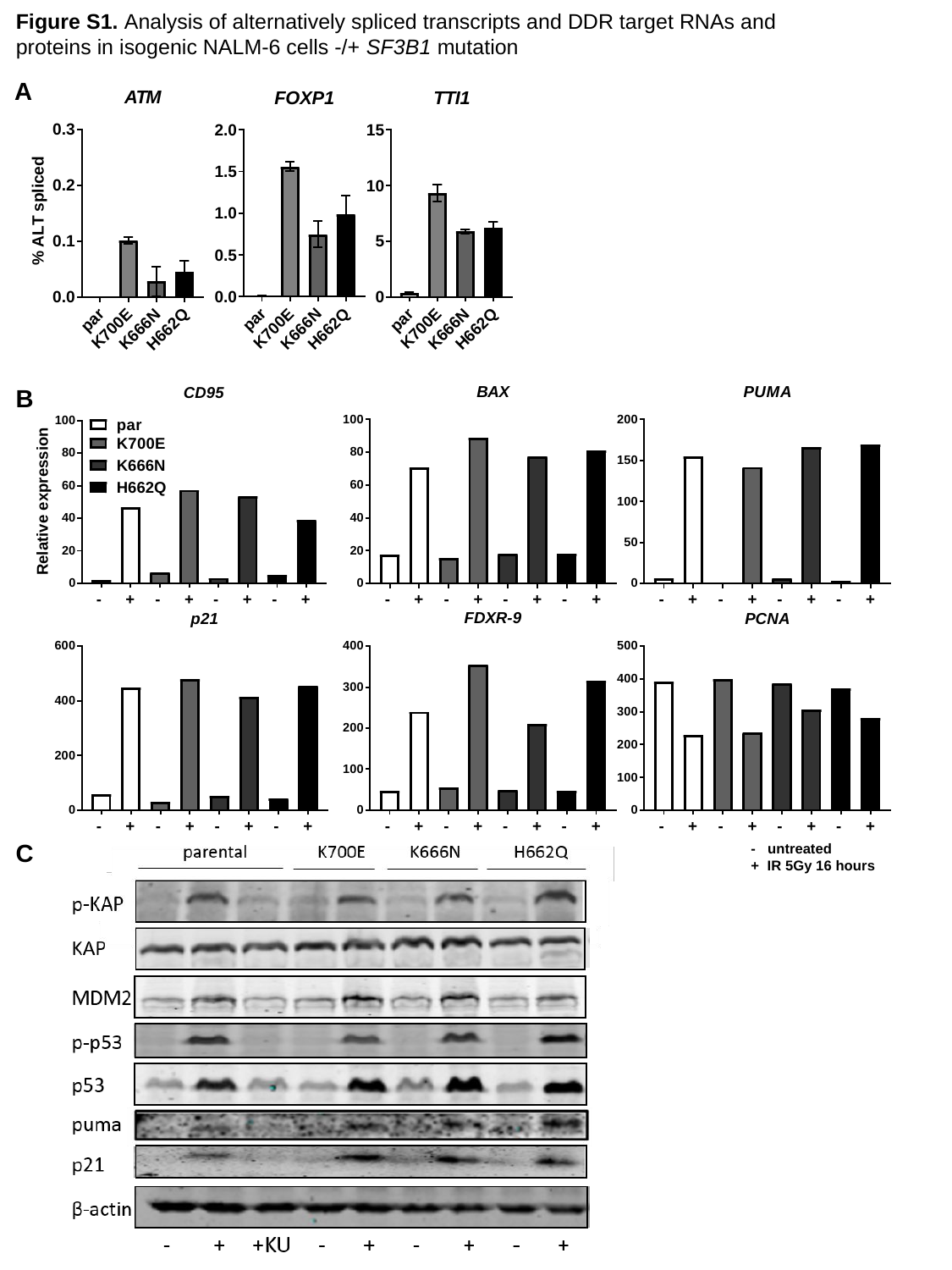

Figure S1. Analysis of alternatively spliced transcripts and DDR target RNAs and proteins in isogenic NALM-6 cells -/+ SF3B1 mutation
A
B
C
- untreated
+ IR 5Gy 16 hours

## Slide 8
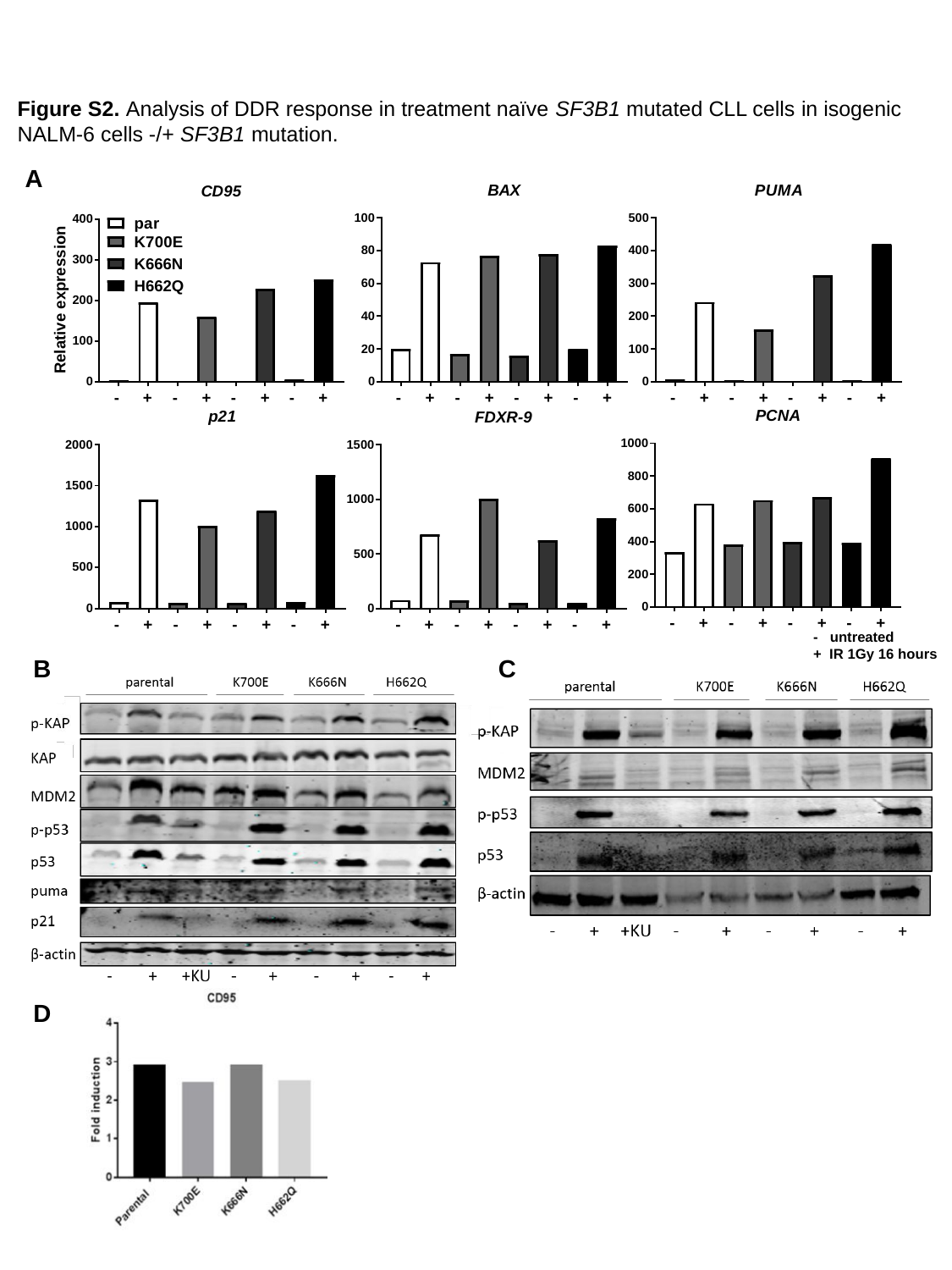

Figure S2. Analysis of DDR response in treatment naïve SF3B1 mutated CLL cells in isogenic NALM-6 cells -/+ SF3B1 mutation.
A
- untreated
+ IR 1Gy 16 hours
B
C
D

## Slide 9
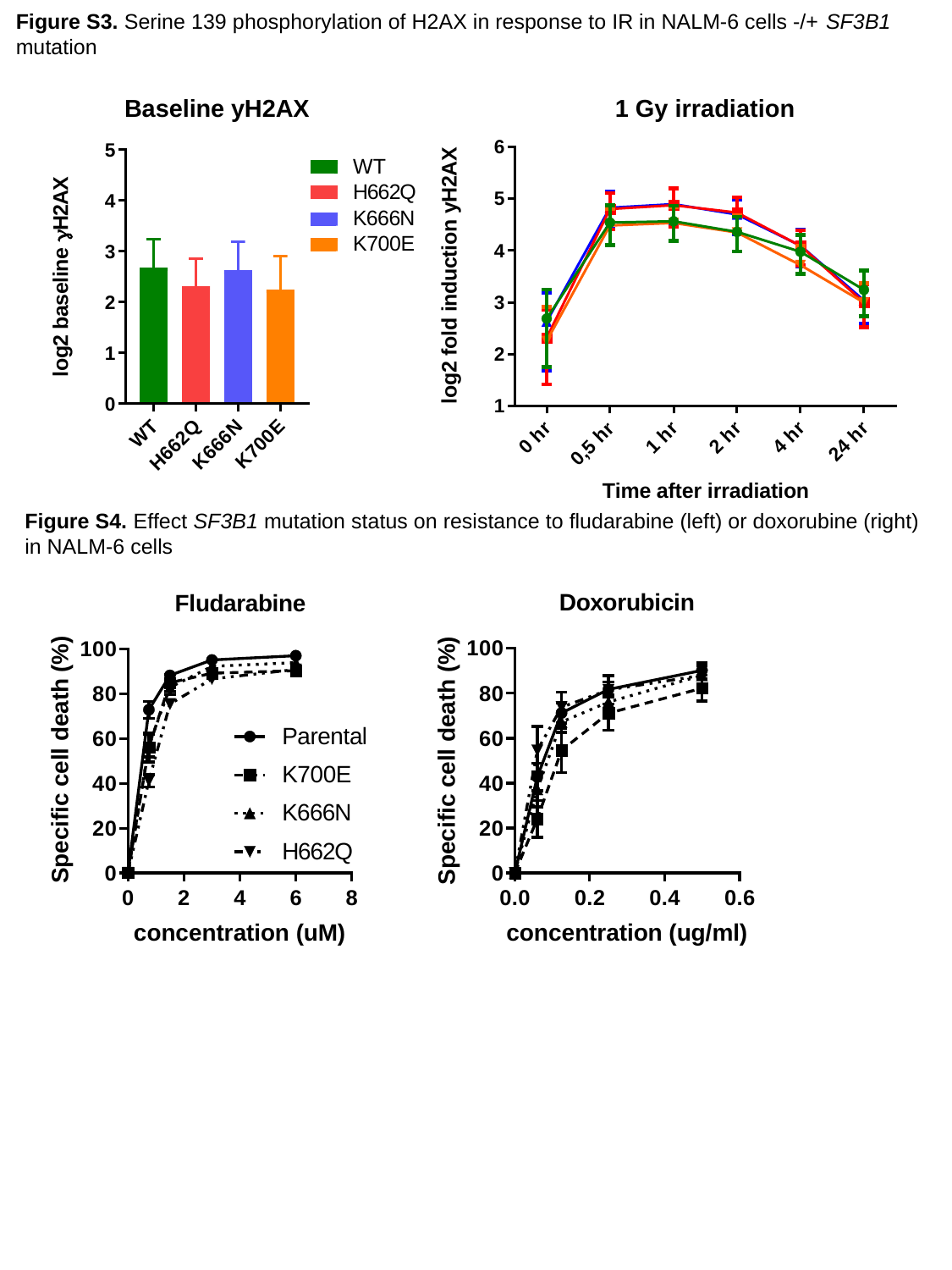

Figure S3. Serine 139 phosphorylation of H2AX in response to IR in NALM-6 cells -/+ SF3B1 mutation
Figure S4. Effect SF3B1 mutation status on resistance to fludarabine (left) or doxorubine (right) in NALM-6 cells

## Slide 10
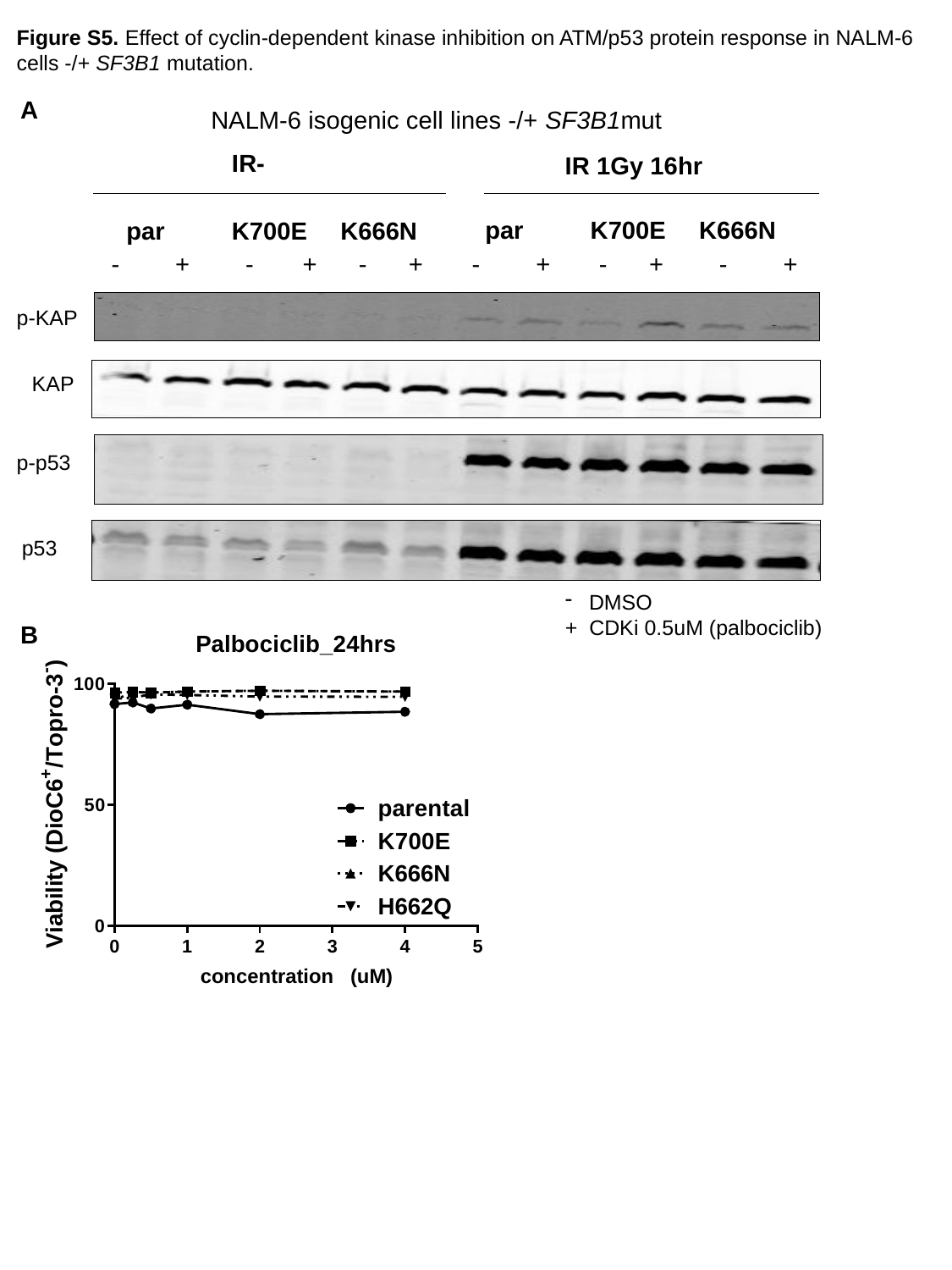

Figure S5. Effect of cyclin-dependent kinase inhibition on ATM/p53 protein response in NALM-6 cells -/+ SF3B1 mutation.
A
NALM-6 isogenic cell lines -/+ SF3B1mut
IR-
IR 1Gy 16hr
K666N
 - + - + - + - + - + - +
par
K700E
par
K666N
K700E
p-KAP
KAP
p-p53
p53
DMSO
+ CDKi 0.5uM (palbociclib)
B
